# Computed tomography in postpartum hemorrhage due to incomplete rupture of an unscarred uterus

**DOI:** 10.1007/s00404-022-06454-y

**Published:** 2022-02-18

**Authors:** Shunya Sugai, Ikunosuke Tsuneki, Toru Yanase, Takumi Kurabayashi

**Affiliations:** Department of Obstetrics and Gynaecology, Niigata City General Hospital, 463-7, Shumoku, Chuo-ku, Niigata, 950-1197 Japan

**Keywords:** Computed tomography, Postpartum hemorrhage, Uterine rupture

## Description

**Fig. 1:**
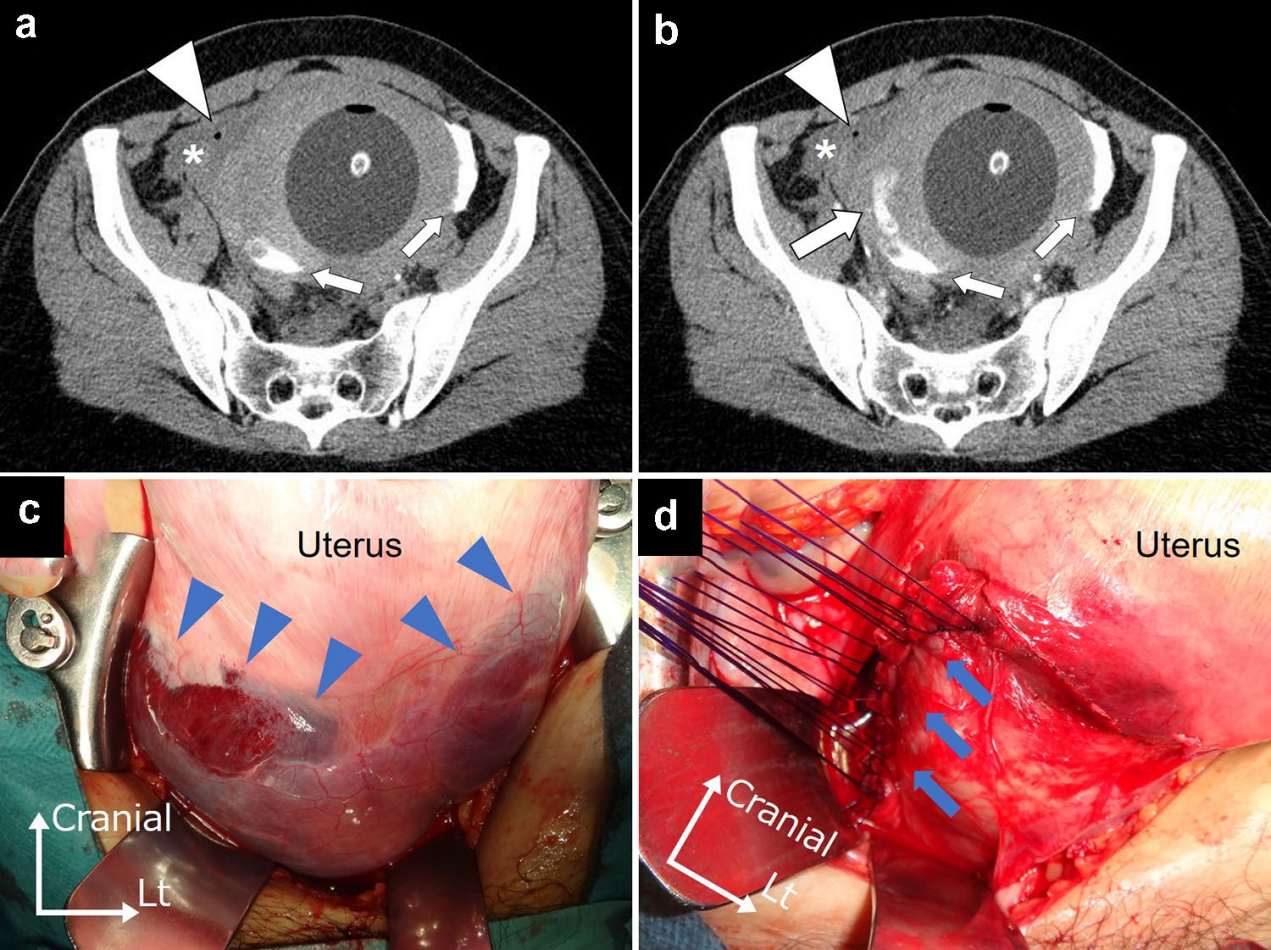
Patient 1: **a** unenhanced and **b** contrast-enhanced computed tomography images obtained during the arterial phase demonstrating extravasation (large arrow). The contrast medium used during uterine artery embolization is visible (small arrows). A hematoma (asterisk) and free air (arrowhead) can be seen in the broad ligament. An intrauterine balloon tamponade device was placed. **c** Hematomas spreading in the broad ligaments (blue arrowheads). **d** The broad ligament was incised, and a 10 cm laceration on the right side of the lower uterus was sutured (blue arrows)

**Fig. 2:**
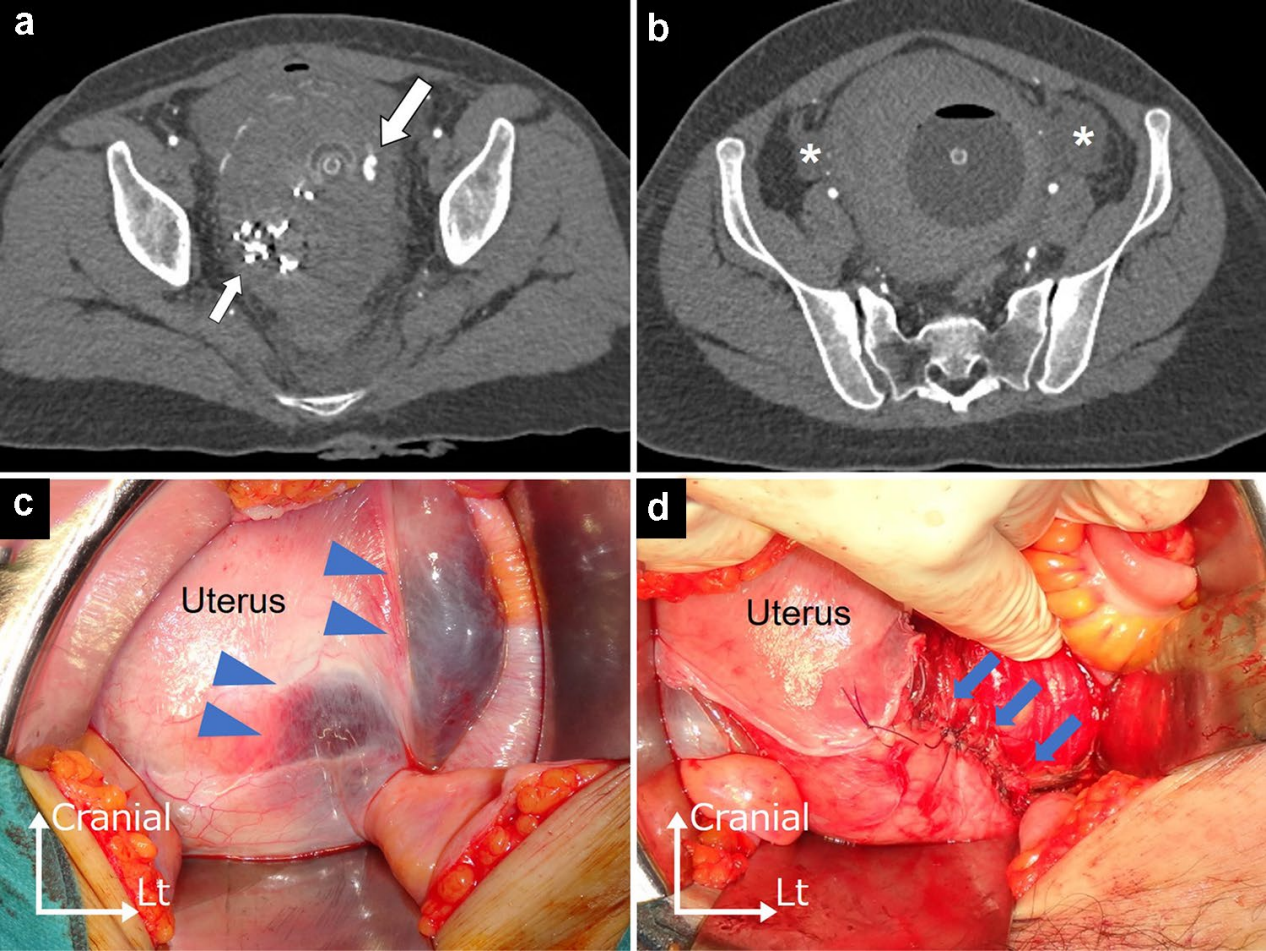
Patient 2: **a**, **b** contrast-enhanced computed tomography images obtained during the arterial phase demonstrating extravasation (large arrow). The small arrow indicates iodoform gauze. A hematoma (asterisks) can be seen in the broad ligament. An intrauterine balloon tamponade device was placed. **c** Hematoma spreading in the broad ligament (blue arrowheads). **d** The broad ligament was incised, and an 8 cm laceration on the left lower uterus was sutured (blue arrows)

Patient 1 was a 33-year-old woman, gravida 2 para 1, who vaginal delivery at 40 weeks’ gestation. Patient 2 was a 37-year-old woman, gravida 2 para 1, who vaginal delivery at 39 weeks’ gestation. Neither case had undergone uterine surgery, including cesarean section. Both patients developed severe postpartum hemorrhage, but not hemorrhagic shock. They did not complain of abdominal pain. Ultrasonography showed heterogeneous echogenicity in the uterus and no hemoperitoneum. Computed tomography showed extravasation from the myometrium and hematoma formation in the broad ligament (Figs. 1a, b and 2a, b). We performed exploratory laparotomy and identified incomplete uterine rupture in both cases; both recovered well with suture repair (Figs. 1c, d and 2c, d).

Uterine rupture is a rare and life-threatening event. Uterine rupture can be divided into complete rupture, which involves all layers of the uterine wall, and incomplete rupture, in which the visceral peritoneum or broad ligament is left intact. Generally, uterine rupture occurs in patients with a scarred uterus, including those with a history of cesarean section; one study showed that uterine rupture occurred in 0.46% of patients with a scarred uterus [1]. Rupture of an unscarred uterus can also occur, but this is even rarer. The reported risk factors for rupture of an unscarred uterus include induction of labor with oxytocin or prostaglandins, breech extraction, age of > 35 years, parity of > 3, and birthweight of > 4000 g [2]. Incomplete rupture of an unscarred uterus is difficult to diagnose, but it can be identified by computed tomography.

## Data Availability

All data related to this report are available from the corresponding author on reasonable request.
